# Lack of Association Between *GBA* Mutations and Motor Complications in European and American Parkinson’s Disease Cohorts

**DOI:** 10.3233/JPD-212657

**Published:** 2021

**Authors:** Jodi Maple-Grødem, Kimberly C. Paul, Ingvild Dalen, Kathie J. Ngo, Darice Wong, Angus D. Macleod, Carl E. Counsell, David Bäckström, Lars Forsgren, Ole-Bjørn Tysnes, Cynthia D.J. Kusters, Brent L. Fogel, Jeff M. Bronstein, Beate Ritz, Guido Alves

**Affiliations:** a The Norwegian Centre for Movement Disorders, Stavanger University Hospital, Stavanger, Norway; b Department of Chemistry, Bioscience and Environmental Engineering, University of Stavanger, Stavanger, Norway; c Department of Neurology, David Geffen School of Medicine, Los Angeles, CA, USA; d Department of Research, Section of Biostatistics, Stavanger University Hospital, Stavanger, Norway; e Clinical Neurogenomics Research Center, David Geffen School of Medicine, University of California, Los Angeles, Los Angeles, CA, USA; f Institute of Applied Health Sciences, University of Aberdeen, Aberdeen, UK; g Department of Clinical Science, Neurosciences, Umeå University, Umeå, Sweden; h Department of Neurology, and Department of Neuroscience, Yale University School of Medicine, New Haven, CT, USA; i Department of Neurology, Haukeland University Hospital, Bergen, Norway; j Department of Clinical Medicine, University of Bergen, Bergen, Norway; k Department of Human Genetics, University of California Los Angeles, Los Angeles, CA, USA; l Department of Epidemiology, UCLA Fielding School of Public Health, Los Angeles, CA, USA; m Department of Biostatistics, UCLA Fielding School of Public Health, Los Angeles, CA, USA; n Department of Neurology, Stavanger University Hospital, Stavanger, Norway

**Keywords:** GBA, Parkinson’s disease, motor complications, dyskinesias, motor fluctuations

## Abstract

**Background:**

Motor complications are a consequence of the chronic dopaminergic treatment of Parkinson’s disease (PD) and include levodopa-induced dyskinesia (LIDs) and motor fluctuations (MF). Currently, evidence is on lacking whether patients with *GBA*-associated PD differ in their risk of developing motor complications compared to the general PD population.

**Objective:**

To evaluate the association of *GBA* carrier status with the development of LIDS and MFs from early PD.

**Methods:**

Motor complications were recorded prospectively in 884 patients with PD from four longitudinal cohorts using part IV of the UPDRS or MDS-UPDRS. Subjects were followed for up to 11 years and the associations of *GBA* mutations with the development of motor complications were assessed using parametric accelerated failure time models.

**Results:**

In 439 patients from Europe, *GBA* mutations were detected in 53 (12.1%) patients and a total of 168 cases of LIDs and 258 cases of MF were observed. *GBA* carrier status was not associated with the time to develop LIDs (HR 0.78, 95% CI 0.47 to 1.26, *p* = 0.30) or MF (HR 1.19, 95% CI 0.84 to 1.70, *p* = 0.33). In the American cohorts, *GBA* mutations were detected in 36 (8.1%) patients and *GBA* carrier status was also not associated with the progression to LIDs (HR 1.08, 95% CI 0.55 to 2.14, *p* = 0.82) or MF (HR 1.22, 95% CI 0.74 to 2.04, *p* = 0.43).

**Conclusion:**

This study does not provide evidence that *GBA*-carrier status is associated with a higher risk of developing motor complications. Publication of studies with null results is vital to develop an accurate summary of the clinical features that impact patients with *GBA*-associated PD.

## INTRODUCTION

The core motor features of Parkinson’s disease (PD) are bradykinesia, rigidity, and tremor. These features are usually improved by treatment with levodopa and patients’ response is well-maintained by intermittent dosing during waking hours, especially in the early stages of disease. However, long-term dopaminergic therapy, particularly with levodopa, often results in the development of motor complications. These are typified by a reduction in the duration and reliability of the treatment related motor improvements, termed motor fluctuations (MF), and the emergence of involuntary movements, termed levodopa-induced dyskinesia (LIDs) [1, 2].

Motor complications impact quality of life and affect the vast majority of patients by 15–20 years of treatment, although the time it takes for them to first develop differs considerably [1, 3, 4]. Several factors have been linked to the development of MF or LIDs, including female sex, younger age, higher motor symptom severity at diagnosis, medication regime, or nonmotor feature severity, including low mood and high anxiety [5–9].

Mutations in the glucocerebrosidase (*GBA*) gene, which encodes the lysosomal enzyme that is deficient in Gaucher’s disease, are important and common risk factors for PD. Patients with *GBA*-associated parkinsonism tend to have an earlier age of onset and develop motor and cognitive impairment faster than the general PD population [10–13]. Recent work has assessed the risk of developing motor complications in *GBA*-associated PD, with conflicting results. The first longitudinal study found no association between *GBA* carrier status and the development of LIDs [14]. Two later studies found that *GBA* carriers were at increased risk of developing LIDs, but their findings regarding the development of MF were inconsistent [9, 15]. Given the importance of the proper recognition of subgroups of patients with PD at increased risk of motor complications, further studies are needed to clarify the impact of *GBA* carrier status on the risk of MF and LIDs.

This study aimed to examine the association between *GBA* carrier status and the development of either MF or LIDs over time in large, well characterized and prospectively followed cohorts of community-based, non-selected patients with PD from Europe and America. Understanding which groups of patients with PD are at high risk for motor complications would be of benefit in terms of prognosis and patient management, and for potential application to clinical trial design.

## MATERIALS AND METHODS

### Study cohorts

In the European dataset, patients with PD were included from the Norwegian ParkWest study [16], the Parkinsonism Incidence in North-East Scotland (PINE) study [17], and the Swedish New Parkinson Patient in Umeå (NYPUM) study [18]. These cohorts provide on-going follow-up of population-based incidence studies of all newly diagnosed PD identified in specific geographic regions, initiated between 2002 and 2009. Briefly, 212 patients were enrolled in the ParkWest study, 211 in the PINE study, and 182 in the NYPUM study with a diagnosis of PD guided by the UK brain bank criteria [19] though not excluding those with a family history of PD. Only those with a confirmed clinical or pathological (if postmortem examination was performed) diagnosis of PD at their latest or final clinical visit were included. Since enrollment, 71 had a diagnosis other than PD during follow-up. Further, 57 declined genotyping, 31 have no available DNA sample or DNA was not extractable, and seven did not consent to follow-up. The remaining 439 patients were eligible for this study. At the time of the study, data from clinical visits for a period of up to ten years were available.

For the American dataset, patients with PD were included from the Parkinson’s Environment Gene (PEG) Study. The PEG cohort provides on-going follow-up of new-onset (up to 5 years after diagnosis) idiopathic PD cases from three rural California counties (Kern, Tulare, Fresno) with participants enrolled in two waves (PEG1 between 2001 and 2007 and PEG2 between 2010 and 2014) [20]. PD patients were all recruited as part of the PEG case-control study through medical groups, neurologists, and public service announcements, and a Parkinson’s disease registry pilot program in these counties. All patients in PEG were seen by movement disorder specialists at least once at baseline, many on multiple occasions and during follow-up, and confirmed as having probable idiopathic PD based on UKBB guidelines except for the family history criterion. Briefly, 849 patients were enrolled in the case-control study, and 525 patients have participated in prospective follow-up visits to assess progression (mean follow-up of 4.7 years (SD = 2.8)). Of those not examined during follow-up (*n* = 324), 174 were deceased (54%), 42 refused or could not be re-contacted (13%), and 108 are pending examinations (33%). Included in the present study were 445 patients who were recruited within 4.0 years of PD diagnosis, assessed for the presence of motor complications, and had genetic information available. At the time of the study, data from up to three clinical assessments were available (maximum follow up 11.4 years from diagnosis).

Studies were approved by respective ethical committees: The Western Norway Regional Committee for Medical and Health Research Ethics, the MultiCentre Research Ethics Committee for Scotland, the Regional Ethics Review Board in Umeå, and the UCLA Institutional Review Board. Written informed consent was signed by all participants.

### Clinical assessments

The clinical assessments have been described in detail before [16–18, 20]. At baseline, general medical and neurological examinations and semi-structured interviews were performed for all participants to establish medical, drug, and family history (first-degree relative with PD, self-reported). Furthermore, all patients were assessed using Hoehn and Yahr staging [21]. At baseline, patients in ParkWest, PINE, NYPUM and PEG1 were assessed using the Unified Parkinson’s Disease Rating Scale (UPDRS) [22] and in PEG2 using the Movement Disorder Society (MDS)-UPDRS [23]. In PINE, ParkWest and NYPUM, patients were examined on PD medications whenever possible, whilst in PEG patients were examined while functionally off PD medications (overnight medication withdrawal) whenever possible for UPDRS part III.

For the European cohorts and PEG1 at baseline, motor complications were detected using UPDRS part IV (motor fluctuations, score ≥ 1 on UPDRS item 36, 37, 38 or 39; and dyskinesias, score ≥ 1 item 32, 33 or 34). For PEG2 and PEG1 follow up visits, motor complications were detected using MDS-UPDRS part IV (motor fluctuations, score ≥ 1 on UPDRS item 4.3 and 4.4; and dyskinesias, score ≥ 1 item 4.1 and 4.2). Home visits were offered in PEG1 to those unable or unwilling to come to the clinic to minimize attrition bias.

Antiparkinsonian treatment was prescribed and adjusted throughout the study by a study neurologist (European dataset) or treating physician (PEG) according to best clinical judgment. We calculated levodopa-equivalent doses (LED) in accordance with published recommendations [24].

### Assessment of GBA status

Genomic DNA was isolated from the peripheral blood of each subject by standard methods. The presence of *GBA* variants in the European dataset has been described in detail [10]: 188 patients of the ParkWest cohort were characterized by whole exome sequencing and six non-synonymous variants were detected (rs76763715/N370S, rs421016/L444P and rs781152868/Y135C, rs2230288/E326K, rs755 48401/T369M, and rs369068553/V460L) and confirmed by direct sequencing of fragments amplified using primers to specifically amplify the functional *GBA* gene and not the pseudogene (Supplementary Table 1) [10]. These variants were genotyped in all available European samples using TaqMan single nucleotide polymorphism genotyping assay (Thermo Fisher Scientific), using ParkWest samples as controls for each detected genotype. The L444P genotype was determined using restriction fragment length polymorphism (PCR-RFLP) assays and all mutations confirmed by direct sequencing of the polymerase chain reaction product (Supplementary Table 1) [25, 26]. For the PEG dataset, all patients were characterized by a TruSeq custom amplicon panel (Illumina Inc., San Diego, CA), using paired probes designed to hybridize to unique target-specific sequences, including the *GBA* gene, and PCR amplification to enrich the target regions. 11 non-synonymous variants were detected in the eligible participants (rs150466109/K-27R, rs14677 4384/R39C, rs144173415/R47Q, rs77834747/I119T, rs409652/G202R, rs78973108/R257Q, rs2230288/E326K, rs75548401/T369M, rs76763715/N370S, rs1064651/D409H, rs421016/L444P) (Supplementary Table 2). All amino acid substitutions are numbered excluding the 39-residue signal peptide.

For the purpose of this study, *GBA* variants were classified based on published reports of pathogenicity in Gaucher’s disease (GD) or reported associations with PD. *GBA* mutations were classified as “severe” (L444P, G202R, R257Q, and D409H) if linked to neuropathic type 1 or 2 Gaucher’s disease (GD) or “mild” (N370S) if associated with the non-neuropathic type 1 GD. “Risk” variants have been reported to increase risk of PD [27, 28] but were linked to GD only when occurring in conjunction with other GBA mutations [29]. The remaining variants are of unknown significance. The initial classification of mutation severity in association with GD was based on a published classification [30] supplemented with more recent evidence from The Human Gene Mutation Database [31] (http://www.hgmd.cf.ac.uk/), CliniVar (https://www.ncbi.nlm.nih.gov/clinvar/), and literature searches (summarized in Supplementary Table 3). Each variant not classified in GD was subsequently assessed for association with PD using literature identified in Pubmed.

### Statistical methods

Participants were identified as *GBA* carriers (with one or more *GBA* mutations) or non-carriers (no *GBA* mutation). For secondary analysis, we further subdivided the *GBA* mutations into severe, mild, risk factor or unknown significance (Supplementary Table 2). Because of the small sample size for the *GBA* mild subgroup, the mild and risk factor categories were combined. Between-group differences were compared using *t*-tests, Mann-Whitney tests and *χ*^2^-tests as appropriate. Non-parametric maximum likelihood estimates (NPMLEs) of the survival distributions were assessed by the expectation-maximization algorithm [32]. Parametric accelerated failure time models were applied for the primary survival analysis, with allowance for interval censoring and with *t* = 0 at time of diagnosis. The Weibull model was deemed optimal (over other parametric models) for time to MF using both the Akaike and the Bayesian information criteria. For LIDs the Weibull model performed similarly to the Gaussian model and was chosen for consistency and interpretability. These comparisons were made for models adjusted for age and sex. Furthermore, log minus log plots were assessed for the unadjusted models and displayed reasonably straight lines. Coefficients from the Weibull model were transformed into hazard ratios (HR), which were presented with 95% confidence intervals (CI). Further adjustment for motor severity at first visit, or ethnicity (American dataset), or repetition of the models using *t* = 0 as time of first visit and including adjustment for disease duration at time of first visit did not affect the effect sizes (data not shown). Cox proportional hazards models assuming right-censored data were applied in secondary analysis to allow for models including time-varying covariates (PD medication) (analyzed only for European data). The time of event was set to the first visit MF or LIDs was recorded and censoring was at the last clinical assessment. There were no substantial differences between the HRs obtained using Cox proportional hazards models and those transformed from the Weibull model in unadjusted or adjusted models. Cox proportional hazards models were next applied adjusting for age and sex and the time varying medication variables total daily LED, use of dopamine agonists, or use of levodopa at each annual visit.

Data preparation, descriptive and between group comparisons were performed in SPSS. NPMLEs of survival distributions, parametric survival analysis for interval-censored data and Cox regression with time-dependent covariates were performed in R v. 4.0.2 with package survival, functions survfit, survreg and coxph. The main plots of survival curves were created with function ggsurvplot of package survminer.

### Data availability

Anonymized data are available on request by any qualified investigator for purposes of replicating procedures and results.

## RESULTS

### Baseline profile of PD-GBA carriers

This study included 884 patients with PD recruited to either one of three European cohorts (ParkWest, PINE or NYPUM; *n* = 439) or the American PEG cohort (*n* = 445). The baseline characteristics of the cohorts are listed in Supplementary Table 4. The median age at diagnosis was 70.7 (14.0) years and 70.0 (13.0) years in the European cohorts and PEG cohort, respectively. The European cohorts comprised 60.8% (267) males and the PEG cohort comprised 61.3% (273) males.

In the European cohorts, *GBA* variants were identified in 53 (12.1%) patients [10] and in the PEG cohorts, *GBA* variants were identified in 36 (8.1%) patients (Table 1). The median age of diagnosis in the *GBA* carrier group was younger (EUR 66.7 years, USA 66.0 years) than in the non-carriers (EUR 71.1 years, USA 70.0 years) and the distributions in the two groups differed significantly in both the European (Mann-Whitney *U p* = 0.01) and PEG cohorts (Mann-Whitney *U p* = 0.03). No further differences were identified between carriers of a *GBA* mutation and non-carriers for demographic or clinical variables assessed at the first clinical visit (Table 1).

### Effect of GBA on the development of motor complications

**Fig. 1.**
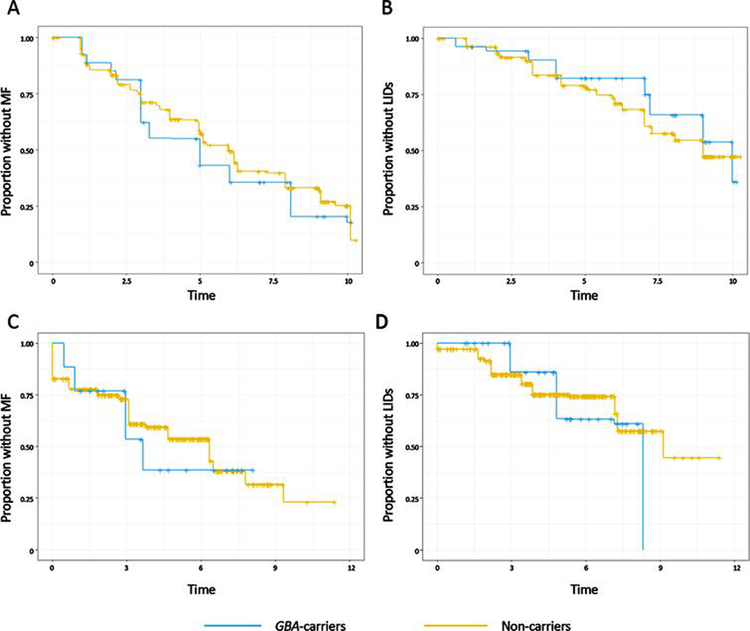
Nonparametric maximum likelihood estimates of the survival distributions for onset of motor fluctuations (MF) and levodopa-induced dyskinesias (LIDs) in the European data set (A and B) or the American dataset (C and D). Time from Parkinson’s disease diagnosis is shown in years. Subjects were grouped into carriers of a *GBA* variant (blue) or non-carriers (yellow).

We first examined the development of motor complications in 439 participants of the European cohorts. Of these, 9 patients were only assessed at one clinical visit and were excluded, leaving 430 patients in the survival analysis. By 10 years of follow-up, 36 (67.9%) of the 53 carriers of any *GBA* mutation had developed MF compared to 222 (58.9%) of the 377 non-carriers, and 18 (34.0%) of 53 *GBA* mutation carriers had developed LIDs compared to 150 (39.8%) of the 377 non-carriers. Parametric accelerated failure time models were applied to assesses the impact of *GBA* carrier status on the time to develop motor complications. The risk of developing MF or LIDs in carriers of a *GBA*-mutation was not different compared to non-carriers in unadjusted analysis (MF: HR 1.22, 95% CI 0.86 to 1.74, *p* = 0.27; LIDs; HR 0.78, 95% CI 0.48 to 1.28, *p* = 0.33) or when controlling for age and sex (Table 2; Fig. 1). Repetition of the model excluding those *GBA* variants of unknown significance did not affect the effect sizes (data not shown). Further, analysis of the impact of either severe *GBA* mutations or mild and risk factor *GBA* mutations on the time to develop motor complications compared to non-carriers revealed no statistically significant association with either MF or LIDs (Table 2). Finally, we applied Cox proportional hazards regression models including time-varying covariates to account for the possible effects of medication regime over the course of PD. Inclusion of time varying total LED, use of levodopa or use of dopamine agonist did not alter the lack of association of *GBA* carrier status with the development of either MF or LIDs (data not shown).

To validate these findings, we next assessed the development of motor complications in 445 participants from the PEG cohort. 440 patients were assessed for MF and 445 patients for LIDs during the study. 10 patients reported MF and 3 LIDs at the time of diagnosis and were excluded from the survival analysis. Parametric accelerated failure time models were then applied to assess the association of *GBA* status with the development of motor complications from the time of PD diagnosis, and showed that in the American cohorts *GBA* -carriers were not at increased risk of developing MF or LIDs in comparison to the non-carriers in unadjusted analysis (MF: HR 1.18, 95% CI 0.72 to 1.96, *p* = 0.51; LIDs: HR 1.08, 95% CI 0.54 to 2.13, *p* = 0.83) or when controlling for age, sex (Table 2, Fig. 1C, D). Similarly, the effect sizes did not change when the analysis was repeated after excluding the variants of unknown significance, or when analysing the impact of either severe *GBA* mutations or mild and risk factor *GBA* mutations compared to non-carriers (data not shown).

## DISCUSSION

In this study we explored the relationship between *GBA* carrier status and the long-term development of motor complications in patients with PD followed prospectively from the early stages of disease. Our findings from both European and American PD populations do not support an association of *GBA* mutations with an increased risk of developing MF or LIDs. These data have important implications of fully understanding the impact of *GBA* mutations on the prognosis of PD.

*GBA*-PD is associated with a younger onset of PD and a more aggressive disease course, including faster progression of motor impairment measured using the UPRDS part III [10–13, 33–35], and it has been suggested that this subgroup of patients may also be at increased risk of developing motor complications. Motor complications negatively affect patients’ quality of life and progression of these symptoms can trigger consideration of advanced treatment options [36]. Further, there is an increasing interest in using genetic stratification to improve the design of clinical trials, with the first trials using *GBA* as an inclusion criterion already completed [37]. Accordingly, it is important to establish which factors put patients at increased risk of developing motor complications.

The majority of previous work assessing motor complications in *GBA*-associated PD is from crosssectional studies. Of seven studies [38–44], none found an association of *GBA*-carrier status with MF and only two [43, 44] with a higher frequency of LIDs at the time of examination. Few longitudinal cohort studies have addressed how motor complications develop over the course of *GBA*-associated PD compared to the general PD population [9, 14, 15]. In agreement with the findings of the current study, two did not show an association between *GBA* carrier status and the time to develop LIDs [14] or MF [9]. Conversely, two studies have reported an association of *GBA* variants and an increased risk of developing LIDs [9] or both LIDs and MF [15] when compared to non-carriers. The first of these from Spain included 532 patients with PD recruited at a late disease stage (on average > 10 years disease duration) and the date of LIDs and MF onset was retrospectively obtained by consulting previous medical records [15]. The Spanish cohort had a similar frequency of *GBA* carriers (12.2%) but a substantially younger age of PD onset (56 ± 12 years) compared to the current study. LIDs were shown to develop earlier in carriers of benign *GBA* variants (in this study including both synonymous and non-synonymous variants) (HR 2.4; 95% CI 1.41 to 4.09; *p* = 0.001) and MF to develop earlier in carriers of either benign *GBA* variants (HR 2.44; 95% CI 1.51 to 3.96; *p* < 0.001) or carriers of severe *GBA* mutations (HR 1.85; 95% CI 1.22 to 2.81; *p* = 0.004) [15]. Surprisingly, in this study the effect on the development of MF was smaller for the more damaging category of *GBA* mutations [15]. Subsequently, a population-based UK study prospectively assessed the development of motor complications in a total of 113 patients and found that whilst *GBA* mutation status missed the threshold for significance in unadjusted analysis (HR 2.75; 95% CI 0.94 to 8.0; *p* = 0.064), *GBA* was associated with the development of LIDs in multivariate analysis adjusted for baseline MMSE score (HR 4.5; 95% CI 1.5 to 13.9: *p* = 0.009) [9]. No associations were found with MF and given the small number of participants and events observed, this study may be too small to draw firm conclusions regarding the association of *GBA* with LIDs or MF. In the present study, in both in the European and American cohorts, *GBA* status had virtually no impact on MF or LIDs with a HR close to one, indicating that patients with *GBA*-associated PD are not at higher risk of developing motor complications.

Despite the small number of studies that have addressed the impact of *GBA* variants on the development of motor complications, there is a striking heterogeneity in their design. For example, the study design (notably retrospective vs prospective follow up and population-based vs specialist clinic settings), cohort size, disease duration at recruitment, length of follow up, methods used to detect motor complications (including UPDRS/MDS-UPDRS part IV or physicians’ diagnosis), and the criteria to select and identify *GBA* variants, were different across the studies identified. Each of these factors can diminish the capacity to compare the effects of *GBA* across different studies and likely contribute to the differences in the findings. This highlights the difficulty in assessing the role of *GBA* variants in PD and advocates for the validation of findings in longitudinal cohort studies designed specifically to study the progression of PD.

Our study had several limitations. The modest number of carriers of individual variants prevented us from analyzing the effect of each variant separately. Further, several cohorts were only screened for selected *GBA* mutations and some individuals with *GBA* variations (particularly severe mutations) were probably missed, which could bias results toward the null. However, the overall frequency of *GBA* variants detected in those cohorts analyzed using targeted genotyping (PINE, 9.4%; NYPUM 15.0%) is similar to those assessed with more comprehensive coverage (PEG, 8.1%; ParkWest, 11.6%) and thus, these biases can be expected to be minor. Similarly, we did not account for other genetic variants, such as in *LRRK2*, that are known to impact motor complications [45, 46] and the course of GBA-associated PD [47]. Furthermore, a number PEG participants presented with either MF or LIDs at their first clinical assessment. This is not unexpected as PEG recruited patients up to five years after PD diagnosis, and studies have shown that motor complications may emerge as early as several months to a few years after the initiation of treatment [48]. Finally, the frequency of visits and the duration of follow up in the PEG cohort was lower than in the European studies, which could result in a lower number of motor complication events detected. Our study also had many important strengths, including the use of large population-representative cohorts and the prospective assessment of motor complications using uniform data-ascertainment methods for more than 4000 study visits analyzed up to 11 years from diagnosis, which address some of the weaknesses of previous studies. Furthermore, both the European and PEG studies made substantial efforts to follow participants until death, including home visits for those no longer willing or able to attend clinic visits, greatly reducing the problem of selection and attrition bias. Finally, we used adjustment for important confounders, including treatment-related factors and were able to validate our findings from the European cohorts in an independent data set from the USA.

## CONCLUSION

In this study we do not find evidence that increased risk of motor complications is a key feature of *GBA*-associated PD but further studies in larger population-based cohorts with comprehensive coverage of GBA variants are needed to further clarify the issue especially with regard to severity of the mutations. The inclusion of negative findings in the narrative of *GBA*-PD is vital to enable a balanced assessment of the clinical features that may differentially affect this subgroup of patients. A clear understanding of the link between *GBA*-PD and MF and LIDs is vital for proper patient management, not least because decisions regarding current treatment for those with *GBA*-PD may be influenced if treatment-related motor complications would be considered as an important side effect among carriers.

## Supplementary Material

Supplement

## Figures and Tables

**Table 1:** Demographic and clinical features of patients with PD

Clinical variables	*European dataset*				*USA dataset*			
	Non-carriers	*GBA* carriers^a^	Severe mutation carriers	Mild mutation and risk variant carriers	Non-carriers	*GBA* carriers^a^	Severe mutation carriers	Mild mutation and risk variant carriers
*N(%)*	386 (87.9)	53 (12.1)	6(1.4)	45 (10.3)	409 (91.9)	36 (8.1)	5(1.1)	29 (6.5)
Male, *N (%)*	233 (60.3)	34 (64.2)	4 (66.7)	28 (62.2)	245 (60.5)	28 (77.8)	3 (60.0)	25 (86.2)
Age at diagnosis	71.1 (13.9)	66.7 (14.2)	63.0(11.0)	68.8 (13.6)	70.0 (14.0)	66.0 (10.0)	66.0 (13.0)	67.0 (9.0)
Positive family history, *N* (%)	49 (12.7)	7 (13.5)	0 (0.0)	7 (15.9)	55 (13.6)	4(11.1)	0 (0.0)	4 (13.8)
Education, y	11.1 (5.0)	11.0(5.5)	11.7 (7.5)	11.1 (5.0)	14.0 (4.0)	13.5 (3.9)	13.6 (4.6)	13.0 (4.0)
UPDRS/MDS-UPDRS III	23.0 (16.5)	21.0(15.5)	18.5 (18.5)	21.0(14.0)	17.0 (14.0)	20.7 (13.8)	17.0(17.3)	20.4 (13.5)
Hoehn & Yahr	2.0 (1.0)	2.0 (1.0)	2.0 (0.6)	2.0 (1.0)	2.0 (0.5)	2.0 (1.5)	2.0 (1.0)	2.0 (1.5)
Duration of PD at baseline visit, y	0.0 (0.1)	0.0 (0.1)	0.0 (0.04)	0.0 (0.1)	1.8 (2.4)	1.2 (2.0)	1.5 (2.7)	1.2 (1.7)

a *GBA* carners include carners of any *GBA* mutation, including those of unknown significance. Values presented as median (IQR) unless stated otherwise. MMSE, Mini-Mental State Examination; PD, Parkinson’s disease, UPDRS III, Unified Parkinson Disease Rating Scale Part III.

**Table 2:** Survival analysis for the development of motor complications

	Progression to MF		Progression to LIDs	
	HR (95% CI)	^*p*^	HR (95% CI)	^*p*^
*European cohorts*				
GBA carrier^†^	1.19 (0.84 to 1.70)	0.33	0.78 (0.47 to 1.26)	0.30
Severe mutation carriers^†^	1.66 (0.68 to 4.07)	0.26	0.33 (0.05 to 2.35)	0.43
Mild mutations and risk factor carriers^†^	1.17 (0.80 to 1.72)	0.41	0.81 (0.49 to 1.36)	0.27
*USA cohorts*				
GBA carrier^†^	1.22 (0.74 to 2.04)	0.43	1.08 (0.55 to 2.14)	0.82
Severe mutation carriers^†^	0.60 (0.08 to 4.41)	0.62	1.36 (0.19 to 10.03)	0.76
Mild mutations and risk factor carriers^†^	1.21 (0.69 to 2.11)	0.51	1.19 (0.57 to 2.46)	0.64

† Non-carrier group used as reference group for statistical analysis. Adjusted for age and sex. CI, confidence interval; HR, hazard ratio: MF, motor fluctuations, LIDs, levodopa induced dyskinesias.

## References

[1] Ahlskog JE, Muenter MD (2001) Frequency of levodoparelated dyskinesias and motor fluctuations as estimated from the cumulative literature. Mov Disord 16, 448–458. 11391738 10.1002/mds.1090

[2] Thanvi B, Lo N, Robinson T (2007) Levodopa-induced-dyskinesia in Parkinson’s disease: Clinical features, pathogenesis, prevention and treatment. Postgrad Med J 83, 384–388. 17551069 10.1136/pgmj.2006.054759 PMC2600052

[3] Manson A, Stirpe P, Schrag A (2012) Levodopa-induced-dyskinesias clinical features, incidence, risk factors, management and impact on quality of life. J Parkinsons Dis 2, 189–198. 23938226 10.3233/JPD-2012-120103

[4] Hely MA, Reid WG, Adena MA, Halliday GM, Morris JG (2008) The Sydney multicenter study of Parkinson’s disease: The inevitability of dementia at 20 years. Mov Disord 23, 837–844. 18307261 10.1002/mds.21956

[5] Bjornestad A, Forsaa EB, Pedersen KF, Tysnes OB, Larsen JP, Alves G (2016) Risk and course of motor complications in a population-based incident Parkinson’s disease cohort. Parkinsonism Relat Disord 22, 48–53. 26585090 10.1016/j.parkreldis.2015.11.007

[6] Scott NW, Macleod AD, Counsell CE (2016) Motor complications in an incident Parkinson’s disease cohort. Eur J Neurol 23, 304–312. 26074125 10.1111/ene.12751

[7] Nicoletti A, Mostile G, Nicoletti G, Arabia G, Iliceto G, Lamberti P, Marconi R, Morgante L, Barone P, Quattrone A, Zappia M (2016) Clinical phenotype and risk of levodopa-induced dyskinesia in Parkinson’s disease. J Neurol 263, 888–894. 26964541 10.1007/s00415-016-8075-6

[8] Kelly MJ, Lawton MA, Baig F, Ruffmann C, Barber TR, Lo C, Klein JC, Ben-Shlomo Y, Hu MT (2019) Predictors of motor complications in early Parkinson’s disease: A prospective cohort study. Mov Disord 34, 1174–1183. 31283854 10.1002/mds.27783 PMC6771533

[9] Kim HJ, Mason S, Foltynie T, Winder-Rhodes S, Barker RA, Williams-Gray CH (2020) Motor complications in Parkinson’s disease: 13-year follow-up of the CamPaIGN cohort. Mov Disord 35, 185–190. 31965629 10.1002/mds.27882 PMC7063985

[10] Lunde KA, Chung J, Dalen I, Pedersen KF, Linder J, Domellof ME, Elgh E, Macleod AD, Tzoulis C, Larsen JP, Tysnes OB, Forsgren L, Counsell CE, Alves G, Maple-Grodem J (2018) Association of glucocerebrosidase polymorphisms and mutations with dementia in incident Parkinson’s disease. Alzheimers Dement 14, 1293–1301. 29792872 10.1016/j.jalz.2018.04.006

[11] Liu G, Locascio JJ, Corvol JC, Boot B, Liao Z, Page K, Franco D, Burke K, Jansen IE, Trisini-Lipsanopoulos A, Winder-Rhodes S, Tanner CM, Lang AE, Eberly S, Elbaz A, Brice A, Mangone G, Ravina B, Shoulson I, Cormier-Dequaire F, Heutink P, van Hilten JJ, Barker RA, Williams-Gray CH, Marinus J, Scherzer CR, HBS; CamPaIGN; PICNICS; PROPARK; PSG; DIGPD; PDBP (2017) Prediction of cognition in Parkinson’s disease with a clinical-genetic score: A longitudinal analysis of nine cohorts. Lancet Neurol 16, 620–629. 28629879 10.1016/S1474-4422(17)30122-9 PMC5761650

[12] Stoker TB, Camacho M, Winder-Rhodes S, Liu G, Scherzer CR, Foltynie T, Evans J, Breen DP, Barker RA, Williams-Gray CH (2020) Impact of GBA1 variants on long-term clinical progression and mortality in incident Parkinson’s disease. J Neurol Neurosurg Psychiatry 91, 695–702. 32303560 10.1136/jnnp-2020-322857 PMC7361014

[13] Maple-Grodem J, Dalen I, Tysnes OB, Macleod AD, Forsgren L, Counsell CE, Alves G (2021) Association of GBA genotype with motor and functional decline in newly diagnosed patients with Parkinson disease. Neurology 96, e1036–e1044. 33443131 10.1212/WNL.0000000000011411 PMC8055329

[14] Oeda T, Umemura A, Mori Y, Tomita S, Kohsaka M, Park K, Inoue K, Fujimura H, Hasegawa H, Sugiyama H, Sawada H (2015) Impact of glucocerebrosidase mutations on motor and nonmotor complications in Parkinson’s disease. Neurobiol Aging 36, 3306–3313. 26422360 10.1016/j.neurobiolaging.2015.08.027

[15] Jesus S, Huertas I, Bernal-Bernal I, Bonilla-Toribio M, Caceres-Redondo MT, Vargas-Gonzalez L, Gomez-Llamas M, Carrillo F, Calderon E, Carballo M, Gomez-Garre P, Mir P (2016) GBA variants influence motor and non-motor features of Parkinson’s disease. PLoS One 11. e0167749. 28030538 10.1371/journal.pone.0167749 PMC5193380

[16] Alves G, Müller B, Herlofson K, HogenEsch I, Telstad W, Aarsland D, Tysnes O-B, Larsen JP (2009) Incidence of Parkinson’s disease in Norway: The Norwegian ParkWest study. J Neurol Neurosurg Psychiatry 80. 851–857. 19246476 10.1136/jnnp.2008.168211

[17] Caslake R, Taylor K, Scott N, Gordon J, Harris C, Wilde K, Murray A, Counsell C (2013) Age-, gender-, and socioeconomic status-specific incidence of Parkinson’s disease and parkinsonism in northeast Scotland: The PINE study. Parkinsonism Relat Disord 19, 515–521. 23462482 10.1016/j.parkreldis.2013.01.014

[18] Linder J, Stenlund H, Forsgren L (2010) Incidence of Parkinson’s disease and parkinsonism in northern Sweden: A population-based study. Mov Disord 25, 341–348. 20108376 10.1002/mds.22987

[19] Daniel SE, Lees AJ (1993) Parkinson’s Disease Society Brain Bank, London: Overview and research. J Neural Transm Suppl 39, 165–172. 8360656

[20] Ritz B, Rhodes SL, Bordelon Y, Bronstein J (2012) alphaSynuclein genetic variants predict faster motor symptom progression in idiopathic Parkinson disease. PLoS One 7, e36199. 22615757 10.1371/journal.pone.0036199 PMC3352914

[21] Hoehn MM, Yahr MD (1967) Parkinsonism: Onset, progression and mortality. Neurology 17, 427–442. 6067254 10.1212/wnl.17.5.427

[22] Fahn S, Elton R, Members of the UPDRS Development Committee (1987) Unified Parkinson’s Disease Rating Scale. In Recent Development in Parkinson’s Disease, Vol. 2, Fahn S, Marsden CD, Caine DB, Goldstein M, eds. Macmillan Health Care Information, Florham Park, NJ.

[23] Goetz CG, Tilley BC, Shaftman SR, Stebbins GT, Fahn S, Martinez-Martin P, Poewe W, Sampaio C, Stern MB, Dodel R, Dubois B, Holloway R, Jankovic J, Kulisevsky J, Lang AE, Lees A, Leurgans S, LeWitt PA, Nyenhuis D, Olanow CW, Rascol O, Schrag A, Teresi JA, van Hilten JJ, LaPelle N, Movement Disorder Society UPDRS Revision Task Force (2008) Movement Disorder Society-sponsored revision of the Unified Parkinson’s Disease Rating Scale (MDS-UPDRS): Scale presentation and clinimetric testing results. Mov Disord 23, 2129–2170. 19025984 10.1002/mds.22340

[24] Tomlinson CL, Stowe R, Patel S, Rick C, Gray R, Clarke CE (2010) Systematic review of levodopa dose equivalency reporting in Parkinson’s disease. Mov Disord 25, 2649 2653. 21069833 10.1002/mds.23429

[25] Stone DL, Tayebi N, Orvisky E, Stubblefield B, Madike V, Sidransky E (2000) Glucocerebrosidase gene mutations in patients with type 2 Gaucher disease. Hum Mutat 15, 181–188. 10649495 10.1002/(SICI)1098-1004(200002)15:2<181::AID-HUMU7>3.0.CO;2-S

[26] Torok R, Zadori D, Torok N, Csility E, Vecsei L, Klivenyi P (2016) An assessment of the frequency of mutations in the GBA and VPS35 genes in Hungarian patients with sporadic Parkinson’s disease. Neurosci Lett 610, 135–138. 26547032 10.1016/j.neulet.2015.11.001

[27] Pankratz N, Beecham GW, DeStefano AL, Dawson TM, Doheny KF, Factor SA, Hamza TH, Hung AY, Hyman BT, Ivinson AJ, Krainc D, Latourelle JC, Clark LN, Marder K, Martin ER, Mayeux R, Ross OA, Scherzer CR, Simon DK, Tanner C, Vance JM, Wszolek ZK, Zabetian CP, Myers RH, Payami H, Scott WK, Foroud T, PD GWAS Consortium (2012) Meta-analysis of Parkinson’s disease: Identification of a novel locus, RIT2. Ann Neurol 71, 370–384. 22451204 10.1002/ana.22687 PMC3354734

[28] Mallett V, Ross JP, Alcalay RN, Ambalavanan A, Sidransky E, Dion PA, Rouleau GA, Gan-Or Z (2016) GBA p.T369M substitution in Parkinson disease: Polymorphism or association? A meta-analysis. Neurol Genet 2, el04. 10.1212/NXG.0000000000000104 PMC5017539 27648471

[29] Hruska KS, LaMarca ME, Scott CR, Sidransky E (2008) Gaucher disease: Mutation and polymorphism spectrum in the glucocerebrosidase gene (GBA). Hum Mutat 29, 567–583. 18338393 10.1002/humu.20676

[30] Beutler E, Gelbart T, Scott CR (2005) Hematologically important mutations: Gaucher disease. Blood Cells Mol Dis 35, 355–364. 16185900 10.1016/j.bcmd.2005.07.005

[31] Stenson PD, Mort M, Ball EV, Chapman M, Evans K, Azevedo L, Hayden M, Heywood S, Millar DS, Phillips AD, Cooper DN (2020) The Human Gene Mutation Database (HGMD((R))): Optimizing its use in a clinical diagnostic or research setting. Hum Genet 139, 1197–1207. 32596782 10.1007/s00439-020-02199-3 PMC7497289

[32] Turnbull BW (1974) Nonparametric estimation of a survivorship function with doubly censored data. J Am Stat Assoc 69, 169–173.

[33] Gan-Or Z, Giladi N, Rozovski U, Shifrin C, Rosner S, Gurevich T, Bar-Shira A, Orr-Urtreger A (2008) Genotype-phenotype correlations between GBA mutations and Parkinson disease risk and onset. Neurology 70. 2277–2283. 18434642 10.1212/01.wnl.0000304039.11891.29

[34] Winder-Rhodes SE, Evans JR, Ban M, Mason SL, Williams-Gray CH, Foltynie T, Duran R, Mencacci NE, Sawcer SJ, Barker RA (2013) Glucocerebrosidase mutations influence the natural history of Parkinson’s disease in a community-based incident cohort. Brain 136, 392–399. 23413260 10.1093/brain/aws318

[35] Liu G, Boot B, Locascio JJ, Jansen IE, Winder-Rhodes S, Eberly S, Elbaz A, Brice A, Ravina B, van Hilten JJ, Cormier-Dequaire F, Corvol JC, Barker RA, Heutink P, Marinus J, Williams-Gray CH, Scherzer CR, International Genetics of Parkinson Disease Progression (IGPP) Consortium (2016) Specifically neuropathic Gaucher’s mutations accelerate cognitive decline in Parkinson’s. Ann Neurol 80. 674–685. 27717005 10.1002/ana.24781 PMC5244667

[36] Dijk JM, Espay AJ, Katzenschlager R, de Bie RMA (2020) The choice between advanced therapies for Parkinson’s disease patients: Why, what, and when? J Parkinsons Dis 10. S65–S73. 32651333 10.3233/JPD-202104 PMC7592668

[37] Mullin S, Smith L, Lee K, D’ Souza G, Woodgate P, Elflein J, Hallqvist J, Toffoli M, Streeter A, Hosking J, Heywood WE, Khengar R, Campbell P, Hehir J, Cable S, Mills K, Zetterberg H, Limousin P, Libri V, Foltynie T, Schapira AHV (2020) Ambroxol for the treatment of patients with Parkinson disease with and without glucocerebrosidase gene mutations: A nonrandomized, noncontrolled trial. JAMA Neurol 77, 427–434. 31930374 10.1001/jamaneurol.2019.4611 PMC6990847

[38] Kumar KR, Ramirez A, Gobel A, Kresojevic N, Svetel M, Lohmann K, C MS, Rolfs A, Mazzulli JR, Alcalay RN, Krainc D, Klein C, Kostic V, Grunewald A (2013) Glucocerebrosidase mutations in a Serbian Parkinson’s disease population. Eur J Neurol 20, 402–405. 22812582 10.1111/j.1468-1331.2012.03817.x

[39] Li Y, Sekine T, Funayama M, Li L, Yoshino H, Nishioka K, Tomiyama H, Hattori N (2014) Clinicogenetic study of GBA mutations in patients with familial Parkinson’s disease. Neurobiol Aging 35, 935 e933–938. 10.1016/j.neurobiolaging.2013.09.019 24126159

[40] Wang C, Cai Y, Gu Z, Ma J, Zheng Z, Tang BS, Xu Y, Zhou Y, Feng T, Wang T, Chen SD, Chan P, Chinese Parkinson Study G (2014) Clinical profiles of Parkinson’s disease associated with common leucine-rich repeat kinase 2 and glucocerebrosidase genetic variants in Chinese individuals. Neurobiol Aging 35, 725 e721–726. 10.1016/j.neurobiolaging.2013.08.012 24095219

[41] Zhang Y, Sun QY, Zhao YW, Shu L, Guo JF, Xu Q, Yan XX, Tang BS (2015) Effect of GBA mutations on phenotype of Parkinson’s disease: A study on Chinese population and a meta-analysis. Parkinsons Dis 2015, 916971. 26421210 10.1155/2015/916971 PMC4572432

[42] Cilia R, Tunesi S, Marotta G, Cereda E, Siri C, Tesei S, Zecchinelli AL, Canesi M, Mariani CB, Meucci N, Sacilotto G, Zini M, Barichella M, Magnani C, Duga S, Asselta R, Solda G, Seresini A, Seia M, Pezzoli G, Goldwurm S (2016) Survival and dementia in GBA-associated Parkinson’s disease: The mutation matters. Ann Neurol 80. 662–673. 27632223 10.1002/ana.24777

[43] Lesage S, Anheim M, Condroyer C, Poliak P, Durif F, Dupuits C, Viallet F, Lohmann E, Corvol JC, Honore A, Rivaud S, Vidailhet M, Durr A, Brice A, French Parkinson’s Disease Genetics Study Group (2011) Large-scale screening of the Gaucher’s disease-related glucocerebrosidase gene in Europeans with Parkinson’s disease. Hum Mol Genet 20, 202–210. 20947659 10.1093/hmg/ddq454

[44] Pulkes T, Choubtum L, Chitphuk S, Thakkinstian A, Pongpakdee S, Kulkantrakorn K, Hanchaiphiboolkul S, Tiamkao S, Boonkongchuen P (2014) Glucocerebrosidase mutations in Thai patients with Parkinson’s disease. Parkinsonism Relat Disord 20, 986–991. 24997549 10.1016/j.parkreldis.2014.06.007

[45] Falla M, Di Fonzo A, Hicks AA, Pramstaller PP, Fabbrini G (2021) Genetic variants in levodopa-induced dyskinesia (LID): A systematic review and meta-analysis. Parkinsonism Relat Disord 84, 52–60. 33561612 10.1016/j.parkreldis.2021.01.020

[46] Marras C, Alcalay RN, Caspell-Garcia C, Coffey C, Chan P, Duda JE, Facheris MF, Fernandez-Santiago R, RuizMartinez J, Mestre T, Saunders-Pullman R, Pont-Sunyer C, Tolosa E, Waro B, LRRK2 Cohort Consortium (2016) Motor and nonmotor heterogeneity of LRRK2-related and idiopathic Parkinson’s disease. Mov Disord 31, 1192–1202. 27091104 10.1002/mds.26614

[47] Ortega RA, Wang C, Raymond D, Bryant N, Scherzer CR, Thaler A, Alcalay RN, West AB, Mirelman A, Kuras Y, Marder KS, Giladi N, Ozelius LJ, Bressman SB, Saunders-Pullman R (2021) Association of dual LRRK2 G2019S and GBA variations with Parkinson disease progression. JAMA Netw Open 4, e215845. 33881531 10.1001/jamanetworkopen.2021.5845 PMC8060834

[48] Fahn S, Oakes D, Shoulson I, Kieburtz K, Rudolph A, Lang A, Olanow CW, Tanner C, Marek K, Parkinson Study Group (2004) Levodopa and the progression of Parkinson’s disease. N Engl J Med 351, 2498–2508. 15590952 10.1056/NEJMoa033447

