# Supplementary material for: Lack of Association Between GBA Mutations and Motor Complications in European and American Parkinson’s Disease Cohorts
Source: J Parkinsons Dis. Author manuscript; Available in PMC 2022 Jan 1. (PMC8609705; doi:10.3233/JPD-212657)
Supplement: Supplement [file NIHMS1756640-supplement-Supplement.pdf]

Supplementary Material

Lack of Association Between *GBA* Mutations and Motor Complications in European and American Parkinson's Disease Cohorts

Supplementary Table 1. Primers used to amplify *GBA* for sequencing

Primer application[a]	Primer 1 (5' to 3')	Primer 2 (5' to 3')
Exons 5-7	CAGGAGCCCAAGTTCCC	AGTTTGGGAGCCAGTCATTT
Exons 8-11	TGTGTGCAAGGTCCAGGATCAG	ACCACCTAGAGGGGAAAGTG
Exons 9-11	GGAGGACCCAATTGGGTGCGT	ACCACCTAGAGGGGAAAGTG

[a] The fragment spanning exons 5-7 was used for sequencing of Y135C, the fragment spanning exons 8-11 was used for sequencing of E326K, T369M, and N370S, and the fragment spanning exons 9 – 11 was used in restriction fragment length polymorphism (PCR-RFLP) assays for the detection and sequencing confirmation of L444P.

Supplementary Table 2. Frequencies of *GBA* variants in each of the cohorts

***GBA* mutation carriers** [a]	**ParkWest**	**PINE**	**NYPUM**	**PEG**
N (TOTAL)	189	117	133	445
Severe mutations				
G202R	-	-	-	1 (0.2)
R257Q	-	-	-	1 (0.2)
D409H	-	-	-	1 (0.2)
L444P	1 (0.5)	1 (0.9)	4 (3.0)	2 (0.4)
Mild mutations				
N370S	1 (0.5)	0	0	3 (0.7)
Risk mutations				
E326K	14 (7.4)	4 (3.4)	11 (8.3)	20 (4.4)
T369M	5 (2.6)	6 (5.1)	5 (3.8)	7 (1.6)
Variants of unknown significance				
K-27R	-	-	-	1 (0.2)
R39C	-	-	-	1 (0.2)
R47Q	-	-	-	1 (0.2)
I119T	-	-	-	1 (0.2)
Y135C	1 (0.5)	0	0	-
V460L	1 (0.5)	0	0	-
***GBA* variant categories** [b]				
non-carriers	167 (88.4)	106 (90.6)	113 (85.0)	409 (91.9)
GBA carriers	22 (11.6)	11 (9.4)	20 (15.0)	36 (8.1)
Severe mutation carriers	1 (0.5)	1 (0.9)	4 (3.0)	5 (1.1)
Mild mutation and risk factor carriers	19 (10.1)	10 (8.5)	16 (12.0)	29 (6.5)

[a] All amino acid substitutions are numbered excluding the 39-residue signal peptide.
[b] "*GBA* carriers" included all patients carrying any of the detected nonsynonymous *GBA* variants. The *GBA* variants were further catagorised as severe, mild or risk mutations or variants of unknown significance.
Two patients carried both E326K and T369M, one both K-27R and D409H, and one both I119T and E326K.
-, PINE and NYPUM were only genotyped for the variants detected by whole exome sequencing in the ParkWest patients.
NC, normal control; NYPUM, the New Parkinson Patient in Umeå project; PD, Parkinson Disease; ParkWest, the Norwegian ParkWest study; PINE, the Parkinsonism Incidence in Northeast Scotland study; PEG, Parkinson's Environment Gene.

Supplementary Table 3. Characteristics of *GBA* variants identified in this study

Position [a]	ID SNP	Allele	Amino acid change [b]	Cohorts [c]	ClinVar [d]	Gaucher disease [e]	Parkinson's disease [f]	Classification [g]
155240707	rs150466109	T>C	K13R / K-27R	USA	Benign	Uncertain [1]	Uncertain [2]	VUS
155239961	rs146774384	G>A	R78C/ R39C	USA	Not found	-	Uncertain [3]	VUS
155239936	rs144173415	C>T	R86Q / R47Q	USA	Not found	-	-	VUS
155238632	rs77834747	A>G	I158T / I119T	USA	Uncertain	Uncertain [4]	-	VUS
155238584	rs781152868	T>C	Y174C / Y135C	EUR	Uncertain	-	-	VUS
155238174	rs409652	C>T	G241R / G202R	USA	Pathogenic	Severe [5-7]		Severe
155237453	rs78973108	C>T	R296Q / R257Q	USA	Pathogenic	Severe [5,6,8]		Severe
155236376	rs2230288	C>T	E365K / E326K	EUR/USA	Benign	Benign [9]	Risk [10]	Risk
155236246	rs75548401	G>A	T408M / T369M	EUR/USA	Benign	Benign [9]	Risk [11]	Risk
155235843	rs76763715	T>C	N409S / N370S	EUR/USA	Pathogenic/Likely pathogenic	Mild [5,12]		Mild
155235727	rs1064651	C>G	D448H / D409H	USA	Pathogenic/Likely pathogenic	Severe [5,13]		Severe
155235252	rs421016	A>C	L483P / L444P	EUR/USA	Pathogenic/Likely pathogenic	Severe [5,14]		Severe
155235205	rs369068553	C>G	V499L / V460L	EUR	Likely pathogenic	-	Uncertain [15]	VUS

[a] Position on chromosome one based on GRCh38.p12

[b] Amino acid change in the full length glucocerebrosidase protein or / the processed form excluding the 39-residue signal peptide.

[c] Cohorts categorized as EUR (European cohorts: ParkWest, PINE or NYPUM) or USA (PEG)

[d] CliniVar classification (ncbi.nlm.nih.gov/clinvar/)[f]

[e] Classification based on severity of Gaucher disease. Classification based on Beutler et al 2005 [30] and supplemented by more recent data from The Human Gene Mutation Database (http://www.hgmd.cf.ac.uk/) [31], CliniVar (https://www.ncbi.nlm.nih.gov/clinvar/) and literature identified in Pubmed (https://pubmed.ncbi.nlm.nih.gov). Updates from Beutler et al 2005 are marked in italic font. -, not reported; uncertain, reported but insufficient evidence to assign pathogenicity.

[f] Each variant not classified in GD was subseqiuently asssessed for association with PD using literature identified in Pubmed. uncertain, reported but insufficient evidence to assign as risk variant.

[g] Categorization of *GBA* variant as severe or benign. See materials and methods for full details.

EUR, Europe; VUS, variants of unknown signiciance

REFERENCES

[1] Rozenberg R, Fox DC, Sobreira E, Pereira LV (2006) Detection of 12 new mutations in Gaucher disease Brazilian patients. *Blood Cells Mol Dis* **37**, 204-209.

[2] Nishioka K, Vilarino-Guell C, Cobb SA, Kachergus JM, Ross OA, Wider C, Gibson RA, Hentati F, Farrer MJ (2010) Glucocerebrosidase mutations are not a common risk factor for Parkinson disease in North Africa. *Neurosci Lett* **477**, 57-60.

[3] Graham OEE, Pitcher TL, Liau Y, Miller AL, Dalrymple-Alford JC, Anderson TJ, Kennedy MA (2020) Nanopore sequencing of the glucocerebrosidase (GBA) gene in a New Zealand Parkinson's disease cohort. *Parkinsonism Relat Disord* **70**, 36-41.

[4] Germain DP, Puech JP, Caillaud C, Kahn A, Poenaru L (1998) Exhaustive screening of the acid beta-glucosidase gene, by fluorescence-assisted mismatch analysis using universal primers: mutation profile and genotype/phenotype correlations in Gaucher disease. *Am J Hum Genet* **63**, 415-427.

[5] Beutler E, Gelbart T, Scott CR (2005) Hematologically important mutations: Gaucher disease. *Blood Cells Mol Dis* **35**, 355-364.

[6] Beutler E, Demina A, Gelbart T (1994) Glucocerebrosidase mutations in Gaucher disease. *Mol Med* **1**, 82-92.

[7] Filocamo M, Mazzotti R, Stroppiano M, Seri M, Giona F, Parenti G, Regis S, Corsolini F, Zoboli S, Gatti R (2002) Analysis of the glucocerebrosidase gene and mutation profile in 144 Italian gaucher patients. *Hum Mutat* **20**, 234-235.

[8] Stone DL, Tayebi N, Orvisky E, Stubblefield B, Madike V, Sidransky E (2000) Glucocerebrosidase gene mutations in patients with type 2 Gaucher disease. *Hum Mutat* **15**, 181-188.

[9] Hruska KS, LaMarca ME, Scott CR, Sidransky E (2008) Gaucher disease: mutation and polymorphism spectrum in the glucocerebrosidase gene (GBA). *Hum Mutat* **29**, 567-583.

[10] Pankratz N, Beecham GW, DeStefano AL, Dawson TM, Doheny KF, Factor SA, Hamza TH, Hung AY, Hyman BT, Ivinson AJ, Krainc D, Latourelle JC, Clark LN, Marder K, Martin ER, Mayeux R, Ross OA, Scherzer CR, Simon DK, Tanner C, Vance JM, Wszolek ZK, Zabetian CP, Myers RH, Payami H, Scott WK, Foroud T, PD GWAS Consortium (2012) Meta-analysis of Parkinson's disease: identification of a novel locus, RIT2. *Ann Neurol* **71**, 370-384.

[11] Mallett V, Ross JP, Alcalay RN, Ambalavanan A, Sidransky E, Dion PA, Rouleau GA, Gan-Or Z (2016) GBA p.T369M substitution in Parkinson disease: Polymorphism or association? A meta-analysis. *Neurol Genet* **2**, e104.

[12] Tsuji S, Martin BM, Barranger JA, Stubblefield BK, LaMarca ME, Ginns EI (1988) Genetic heterogeneity in type 1 Gaucher disease: multiple genotypes in Ashkenazic and non-Ashkenazic individuals. *Proc Natl Acad Sci U S A* **85**, 2349-2352.

[13] Filocamo M, Grossi S, Stroppiano M, Tortori-Donati P, Regis S, Allegri A, Di Rocco M (2005) Homozygosity for a non-pseudogene complex glucocerebrosidase allele as cause of an atypical neuronopathic form of Gaucher disease. *Am J Med Genet A* **134A**, 95-96.

[14] Tsuji S, Choudary PV, Martin BM, Stubblefield BK, Mayor JA, Barranger JA, Ginns EI (1987) A mutation in the human glucocerebrosidase gene in neuronopathic Gaucher's disease. *N Engl J Med* **316**, 570-575.

[15] McNeill A, Duran R, Hughes DA, Mehta A, Schapira AH (2012) A clinical and family history study of Parkinson's disease in heterozygous glucocerebrosidase mutation carriers. *J Neurol Neurosurg Psychiatry* **83**, 853-854.

Supplementary Table 4. Summary of the cohorts included in this study

	European cohorts				American cohorts		
	**ParkWest**	**PINE**	**NYPUM**	**Combined**	**PEG1**	**PEG2**	**Combined**
N total	189	117	133	439	219	226	445
Male, N (%)	115 (60.8)	72 (61.5)	80 (60.2)	267 (60.8)	124 (57.7)	149 (65.9)	273 (61.3)
Age at diagnosis, y	68.5 (12.4)	73.3 (12.7)	71.4 (14.4)	70.7 (14.0)	69.0 (14.0)	70.0 (13.0)	70.0 (13.0)
Family history, N (%)	16 (13.7)	16 (13.7)	21 (11.1)	56 (12.8)	24 (11.2)	35 (15.5)	59 (13.3)
Education, y	11.0 (5.0)	11.0 (5.0)	8.0 (5.0)	11.0 (5.0)	13.0 (4.0)	14.0 (5.0)	14.0 (4.0)
UPDRS III	21.0 (16.5)	23.0 (15.0)	26.0 (15.0)	23.0 (16.0)	18.0 (13.0)	17.0 (16.0)	18.0 (14.0)
Hoehn & Yahr	2.0 (1.0)	2.5 (1.0)	2.0 (0.5)	2.0 (1.0)	2.0 (1.0)	2.0 (0.5)	2.0 (0.5)
Duration of PD at baseline, y	0.01 (0.1)	0.01 (0.1)	0.1 (0.1)	0.1 (0.1)	0.7 (1.8)	2.6 (1.7)	1.7 (2.4)
Duration of follow up from diagnosis, y	9.1 (2.3)	8.0 (5.1)	8.0 (4.9)	9.0 (4.1)	5.6 (2.7)	4.7 (2.2)	5.1 (2.5)
Number of deaths, n (%)	66 (34.9)	69 (59.0)	59 (44.4)	194 (44.2)	100 (46.5)	22 (9.7)	122 (27.7)
Time to death, y, mean (SD)	8.2 (3.3)	6.9 (3.3)	6.7 (2.7)	7.4 (3.3)	7.6 (2.6)	5.6 (1.8)	7.2 (2.6)

Values presented as median (IQR) unless stated otherwise.
PD, Parkinson's disease, SD, standard deviation, UPDRS III, Unified Parkinson Disease Rating Scale Part III.
